# Antibiotic resistance of ESKAPE group-microorganisms in health institutions from Hermosillo and Ciudad Obregón, Sonora, México

**DOI:** 10.3389/fcimb.2024.1348093

**Published:** 2024-03-07

**Authors:** Maritza Lizeth Álvarez-Ainza, Pedro Alejandro Fong-Coronado, Eduardo Ruiz-Bustos, Lucía Guadalupe Castillón-Campaña, Idania Emedith Quintero-Reyes, Luis Armando Duarte-Zambrano, Enrique Bolado-Martínez

**Affiliations:** ^1^ Laboratorio de Microbiología, Departamento de Ciencias Químico-Biológicas, Universidad de Sonora, Hermosillo, Sonora, Mexico; ^2^ Laboratorio de Análisis Especializantes, Departamento de Ciencias de la Salud, Universidad de Sonora, Ciudad Obregón, Sonora, Mexico; ^3^ Centro de Investigación Sobre Enfermedades Infecciosas (CISEI) Instituto Nacional de Salud Pública, Cuernavaca, Morelos, Mexico

**Keywords:** ESKAPE, antibiotic resistance, clinical isolates, Sonora, México

## Abstract

**Introduction:**

*Enterococcus faecium, Staphylococcus aureus*, *Klebsiella pneumoniae, Acinetobacter baumannii, Pseudomonas aeruginosa*, and *Enterobacter* spp. are microorganisms referred as the ESKAPE group pathogens. These microorganisms have generated great concern in health institutions around the world since most of them have resistance to multiple antibiotics and cause most infections associated with healthcare, as well as community infections. The aim of this study was the analysis of antibiotic resistance in microorganisms of the ESKAPE group, recovered from clinical samples in 11 health institutions from Hermosillo and Ciudad Obregón in the State of Sonora, México, during the period from 2019 to 2020.

**Methods:**

A cross-sectional, descriptive, observational, and temporality epidemiological study was carried out. A comparative and statistical analysis of antibiotic resistance was carried out using the chi-square test, and small values were analyzed using Fisher’s exact test p ≤ 0.05.

**Results and discussion:**

All the ESKAPE group microorganisms showed significant differences in antibiotic resistance percentages between both cities. High resistance percentages for some antibiotics, like cephalosporins and ciprofloxacin were detected for *Klebsiella pneumoniae* and *Acinetobacter baumannii*.

## 1 Introduction

Antibiotic resistance has become a public health problem, with high morbidity and mortality rates affecting mainly countries with emerging economies (Zhen et al., 2019). The World Health Organization (WHO) considers that in 2050, infections associated to Antimicrobial Resistance (AMR) will be responsible for 10 million deaths per year (Giono-Cerezo et al., 2020). The Centers for Disease Control and Prevention (CDC) estimate that in the United States of America (USA), infections related to antibiotic-resistant microorganisms are responsible for at least 23,000 deaths per year (Yu-Xuan et al., 2020). Due to the infections caused by AMR bacteria, health personal needs to use high toxicity antibiotics, like colistin, or some of a limited list of the last generation antimicrobials (Benkő et al., 2020).

In February 2017, the WHO published a list of antibiotic-resistant microorganisms for which the development of new antimicrobial treatments is considered urgent. This list includes microorganisms from the ESKAPE group: *Enterococcus faecium, Staphylococcus aureus, Klebsiella pneumoniae, Acinetobacter baumannii, Pseudomonas aeruginosa*, and *Enterobacter* spp (De Oliveira et al., 2020). This group of microorganisms is highly relevant due to their intrinsic and extensive antibiotic resistance, as well as being capable of acquiring multiple genes that confer them multidrug resistance (Ayobami et al., 2022). Also, they are considered the cause of most healthcare-associated infections (HAIs), especially for severely ill and immunocompromised patients (Yu-Xuan et al., 2020; Ayobami et al., 2022). Several studies show that patients with AMR infections are more difficult to receive an adequate treatment that allow them to resolve the infection, allowing to spread antimicrobial resistance, but also, this situation entails that those patients, are more likely to be admitted in the ICU, and be taken more antibiotic treatment (Zhen et al., 2019; Santos-Zonta et al., 2020).

Locally there are no studies or reports that evaluate the resistance to antimicrobials of the ESKAPE group. During 2014-2015, a surveillance study of bacterial resistance was carried out in six health institutions in the city of Hermosillo, México, in which antibiotic resistance was evaluated. The results highlight the high percentages of resistance to ampicillin/sulbactam, and resistance to tigecycline, in *K. pneumoniae* isolates. The study also showed low susceptibility to cefepime, aztreonam, meropenem, ciprofloxacin, amikacin, gentamicin, and tobramycin in *P. aeruginosa* isolates (Bolado-Martínez et al., 2018).

Due to these previous results, it is important to implement measures that include active epidemiological surveillance to obtain more information regarding the prevalence and resistance of microorganisms of the ESKAPE group in health institutions in Sonora. This will allow timely detection of microorganisms of the ESKAPE group, to identify their antibiotic resistance profiles and to use oportunally the antibiotics that each patient require. The objective of this study was to analyze the antibiotic resistance of ESKAPE group microorganisms, recovered from clinical samples in 11 health institutions in Hermosillo and Ciudad Obregón, Sonora, México during the 2019-2020 period.

## 2 Materials and methods

### 2.1 Study description

A cross-sectional, descriptive, observational and temporality epidemiological study was carried out (Hernández, 2017). An intentional non-probabilistic sample was used without a specific age interval (Hernández-Ávila and Carpio, 2019). The data, obtained from automated microbiology systems (Vitek2, BioMerieux, or Phoenix, Becton Dickinson), was obtained using the BacLink free software (https://amrtracker.com/whonet/baclink.html). Tha BacLink databases were converted in a WHONET database (Agarwal et al., 2009), that allowed the observation of the frequency of isolated microorganisms, their susceptibility patterns, as well as the analysis of the results of antibiotic resistance. Data collection was carried out in 11 health institutions in the cities of Hermosillo and Ciudad Obregón, Sonora, México between July 1, 2019, and June 30, 2020. Similarly, between July 1, 2014, and June 30, 2015; data was collected from six health institutions of the city of Hermosillo.

### 2.2 Statistical analysis

A comparative analysis of antibiotic resistance was carried out between health institutions in Hermosillo and Ciudad Obregón, during the period 2019-2020. Comparative analyzes were carried out between the antibiotic resistance results for health institutions in Hermosillo during the periods 2014-2015 and 2019-2020. Statistical analyzes were carried out using the chi-square test, using a significance value of 0.05 or less in both tails. When values obtained were too small, Fisher’s exact test was used, considering the same significance value. The statistical analysis was carried out in Microsoft Excel.

## 3 Results

### 3.1 ESKAPE group microorganisms in clinical samples

During the 2019-2020 period, 4,545 isolates of microorganisms belonging to the ESKAPE group were identified. As shown in **Table 1**, the highest number of microorganisms were recovered from urine samples.

**Table 1:** Microorganisms of the ESKAPE group and clinical samples from which they were recovered at health institutions in Hermosillo and Ciudad Obregón, Sonora.

Sample	Isolates	Percentage
Urine	1180	26
Bronchial	632	14
Blood	516	11.3
Pharynx	386	8.5
Wound	305	6.7
Secretion	219	4.8
Sputum	206	4.5
Others	197	4.3
Unspecified	151	3.3
Catheter	147	3.2
Stool	95	2.1
Vagina	85	1.8
**Total**	**4545**	

### 3.2 ESKAPE group microorganism distribution

*Klebsiella pneumoniae* was the ESKAPE group microorganism recovered in the highest proportion, with 1,320 (29.0%) isolates, followed by *Pseudomonas aeruginosa* (1,206, 26.5%), *Staphylococcus aureus* (1,028, 22.6%), *Acinetobacter baumannii* (458, 10.0%), *Enterobacter* spp. (442, 9.6%) and *Enterococcus faecium* (93 2.0%) clinical isolates. The *Enterobacter* species identified were *E. cloacae* (437), *E. asburiae* (2), *E. gergoviae* (2) and *E. hormaechei* (1).

Most of the microorganisms (3,273) were recovered in health institutions from Hermosillo. The study identified *K. pneumoniae* in 920 (28.1%), *P. aeruginosa* in 914 (28.0%), *S. aureus* in 807 (24.6%), *Enterobacter* sp. in 320 (9.7%), *A. baumannii* in 241 (7.3%) and *E. faecium* in 71 (2.0%) clinical isolates. In Ciudad Obregón, 1,272 isolates from the ESKAPE group were obtained, 400 (31.4%) corresponded to *K. pneumoniae*, 292 (23%) to *P. aeruginosa*, 221 (17.0%) to *S. aureus*, 217 (17.0%) to *A. baumannii*, 120 (9.4%) to *Enterobacter* spp., and 22 (1.7%) to *Enterococcus faecium*.

**Figure 1:**
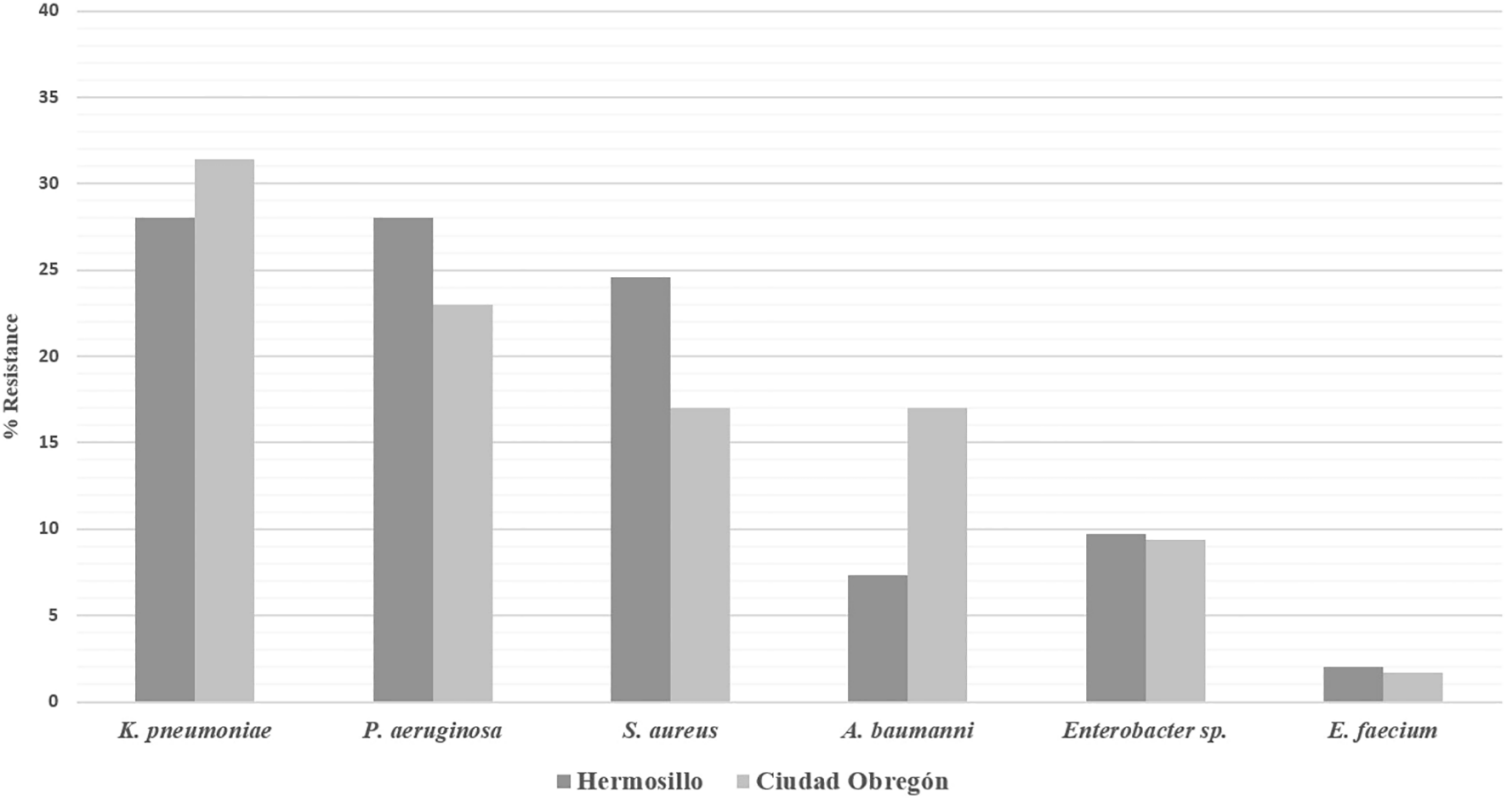
Comparison between ESKAPE microorganisms recovered in 8 health institutions in Hermosillo and 3 institutions in Ciudad Obregón, Sonora (2019–2020).

**Figure 1** shows that percentages of microorganisms isolated from Hermosillo and Ciudad Obregón did not show significant variability between the two cities, except in the case of *A. baumannii*, where a greater number of isolates of this microorganism can be observed in three institutions from Ciudad Obregón, compared to the eight institutions from Hermosillo, Sonora that participated in this study. Those results must be considered, since *A. baumannii* represented the 17% (217 of 1,272 clinical isolates) from the ESKAPE organisms in Ciudad Obregón, while only the 7.4% (241 of 3,273 clinical isolates) from the ESKAPE organisms in Hermosillo.

### 3.3 Antibiotic resistance during the 2019-2020 period

#### 3.3.1 Enterococcus faecium

As shown in **Table 2**, *E. faecium* isolates showed less than 40% resistance to fluoroquinolones (ciprofloxacin, levofloxacin). No susceptibility to aminoglycosides was detected and there was no significant difference in vancomycin resistance, between Hermosillo and Ciudad Obregón institutions; however, a greater number of isolates were reported in Hermosillo. There is a high percentage of strains with resistance to tetracyclines, especially in Hermosillo, where difference is significant compared to Ciudad Obregón.

**Table 2:** Percentages of antibiotic resistance in ESKAPE group clinical isolates, recovered in the 2019-2020 period at 11 health institutions from Hermosillo and Ciudad Obregón, Sonora.

*E. faecium*			
Antibiotics	% R Hermosillo	% R Cd. Obregón	*p*
Ampicillin	47	43	0.76
Ciprofloxacin	34	38	0.71
Streptomycin-H	0	0	1
Gentamicin-H	0	0	1
Levofloxacin	26	29	0.82
Linezolid	6	5	0.87
**Tetracycline**	**74**	**48**	0.02*
Vancomycin	15	0	0.06
*S. aureus*			
**Ciprofloxacin**	**9**	**26**	0.0001*
**Clindamycin**	**21**	**33**	0.0002*
Daptomycin^φ^	6	7	0.63
**Erythromycin**	**20**	**33**	0.0001*
Gentamicin	6	8	0.26
**Levofloxacin**	**7**	**26**	0.0001*
Linezolid	2	2	0.54
**Moxifloxacin**	**6**	**24**	0.0001*
Nitrofurantoin	1	1	0.51
**Oxacillin**	**17**	**29**	0.0001*
Tetracyclin	5	4	0.45
**TMP/SXT**	**3**	**7**	0.01*
Vancomycin	9	6	0.19
*Acinetobacter baumannii*			
**Ampicillin/Sulbactam**	**39**	**56**	0.0004*
**Cefepime**	**60**	**81**	0.0001*
**Cefotaxime**	**63**	**81**	0.0001*
**Ceftazidime**	**62**	**81**	0.0001*
**Ceftriaxone**	**64**	**81**	0.0001*
**Ciprofloxacin**	**60**	**81**	0.0001*
**Gentamicin**	**33**	**42**	0.02*
**Meropenem**	**53**	**79**	0.0001*
**TMP/SXT**	**60**	**83**	0.0001*
*E. cloacae*			
Amikacin	8	8	0.84
Cefepime	16	14	0.59
Cefotaxime	31	31	0.94
Ceftazidime	29	29	0.95
Ceftriaxone	34	32	0.64
Cefuroxime	37	36	0.95
Cefuroxime axetil	37	36	0.95
Ciprofloxacin	25	25	0.95
Ertapenem	10	10	0.87
Gentamicin	16	11	0.25
Meropenem	6	6	0.95
**Nitrofurantoin**	**20**	**32**	0.01*
TMP/SXT	24	24	0.92
*K. pneumoniae*			
Amikacin	4	4	0.93
**Ampicilina/Sulb**	**34**	**48**	0.0001*
Cefepime	14	15	0.36
**Cefotaxime**	**31**	**47**	0.0001*
Amikacin	4	4	0.93
**Ceftazidime**	**22**	**31**	0.0001*
**Ceftriaxone**	**31**	**47**	0.0001*
**Cefuroxime**	**34**	**51**	0.0001*
**Cefuroxime axetil**	**34**	**51**	0.0001*
**Ciprofloxacin**	**34**	**51**	0.0001*
**Ertapenem**	**3**	**6**	0.001*
**Gentamicin**	**18**	**36**	0.0001*
**Meropenem**	**2**	**5**	0.003*
**Nitrofurantoin**	**21**	**29**	0.0009*
**TMP/SXT**	**38**	**45**	0.01*
*Pseudomonas aeruginosa*			
**Amikacin**	**21**	**32**	0.0006*
**Cefepime**	**19**	**31**	0.0001*
**Ceftazidime**	**23**	**35**	0.0001*
**Ciprofloxacin**	**30**	**41**	0.0008*
**Gentamicin**	**14**	**21**	0.006*
Meropenem	34	38	0.12

% R, Resistance percentage; Streptomycin-H y Gentamicin-H, High-level resistance to aminoglycosides. ^φ^For daptomycin, *S. aureus* isolates are classified as non-susceptible. *p ≤ 0.05 indicates statistical difference (bold Font). TMP/SXT, Trimethoprim/sulfamethoxazole.

#### 3.3.2 Staphylococcus aureus

Low levels of resistance to fluoroquinolones are observed in health institutions in Hermosillo, in contrast to Ciudad Obregón, where there are high percentages of resistance, being statistically significant between both cities (**Table 2**). Susceptibility to commonly used broad-spectrum antibiotics such as clindamycin, ciprofloxacin, and trimethoprim/sulfamethoxazole is also observed.

#### 3.3.3 Klebsiella pneumoniae

**Table 2** shows that, except for cefepime, statistically significant differences were observed in the percentages of resistance for all β-lactams between both cities. In Ciudad Obregón institutions, approximately 50% of the *K. pneumoniae* clinical isolates showed resistance to all β-lactams, excluding fourth generation cephalosporins and carbapenems. Meanwhile, in Hermosillo susceptibility to β-lactams did not exceed 40%. In Ciudad Obregón there are high levels of resistance to ciprofloxacin, trimethoprim/sulfamethoxazole, gentamicin, and nitrofurantoin.

#### 3.3.4 Acinetobacter baumannii

A statistically significant difference was identified in the percentages of resistance to antibiotics obtained for *A. baumannii* clinical isolates from Hermosillo and Ciudad Obregón (**Table 2**). Cephalosporins and carbapenems do not seem to be a first-choice treatment option for infections caused by this microorganism, mainly in Ciudad Obregón. In both cities, a marked decrease in resistance to β-lactams is observed when a β-lactamase inhibitor such as sulbactam is used. A high proportion of the isolates showed resistance to ciprofloxacin, trimethoprim/sulfamethoxazole, and gentamicin.

#### 3.3.5 Pseudomonas aeruginosa

Regarding *P. aeruginosa* isolates, the results of this study showed higher percentages of resistance in Ciudad Obregón and most of the differences are statistically significant (**Table 2**). As with *A. baumannii*, there is a widespread problem of *P. aeruginosa* AMR isolates in Ciudad Obregón institutions.

#### 3.3.6 Enterobacter cloacae

No statistically significant differences were observed in antibiotic resistance for *E. cloacae* clinical isolates recovered in Hermosillo and Ciudad Obregón (**Table 2**). However, it should not be ignored that resistance to second and third generation cephalosporins is high. The difference between resistance to nitrofurantoin is significant and is greater in Ciudad Obregón than Hermosillo.

### 3.4 Antibiotic resistance during the 2014-2015 and 2019-2020 periods

#### 3.4.1 Enterococcus faecium

As shown in **Table 3**, resistance to fluoroquinolones in *E. faecium* decreased significantly by 2019-2020 in contrast to 2014-2015. Another result that stands out is the reduction to 0% of high-level resistance to aminoglycosides. During 2014-2015, there was no resistance to linezolid, however by 2019-2020, just four years later, its increase is notable. Similarly, the high increase in resistance to tetracycline is a matter of concern.

**Table 3:** Percentages of antibiotic resistance in ESKAPE group clinical isolates, recovered in the 2014-2015 and 2019-2020 periods at 8 health institutions from Hermosillo, Sonora.

*E. faecium*			
Antibiotics	% R 2014-2015	% R 2019-2020	*p*
Ampicillin	46	48	0.85
**Ciprofloxacin**	**47**	**25**	0.03*
**Gentamicin-H**	**25**	**0**	0.001*
**Levofloxacin**	**37**	**17**	0.03*
Linezolid	0	4	0.5
**Tetracycline**	**42**	**71**	0.007*
Vancomycin	3	6	0.44
*S. aureus*			
**Ciprofloxacin**	**12**	**8**	0.009*
**Clindamycin**	**29**	**20**	0.0001*
**Daptomycinφ**	**12**	**6**	0.009*
Erythromycin	24	21	0.17
Gentamicin	4	5	0.47
Levofloxacin	10	18	0.27
Linezolid	2	2	0.67
**Moxifloxacin**	**4**	**7**	0.008*
Nitrofurantoin	2	2	0.43
**Oxacillin**	**12**	**16**	0.04*
**Tetracycline**	**8**	**6**	0.09*
**TMP/SXT**	**8**	**2**	0.0001*
Vancomycin	9	11	0.49
*Acinetobacter baumannii*			
Ampicillin/Sulbactam	35	38	0.69
Cefepime	32	42	0.23
**Cefotaxime**	**100**	**47**	0.0002*
Ceftazidime	71	45	0.07
Ceftriaxone	38	41	0.77
Ciprofloxacin	34	43	0.33
Gentamicin	34	34	0.95
**Meropenem**	**75**	**28**	0.0001*
TMP/SXT	40	41	0.96
*E. cloacae*			
Amikacin	11	6	0.18
**Cefepime**	**26**	**9**	0.0009*
**Cefotaxime**	**48**	**21**	0.002*
**Ceftazidime**	**45**	**23**	0.01*
Ceftriaxone	39	28	0.07
**Cefuroxime**	**77**	**28**	0.0001*
Cefuroxime axetil	19	15	0.47
Ciprofloxacine	7	10	0.47
**Ertapenem**	**22**	**5**	0.0001*
Gentamicina	12	8	0.28
Meropenem	24	20	0.48
**Nitrofurantoin**	**29**	**11**	0.001*
TMP/SXT	11	6	0.18
*K. pneumoniae*			
Amikacine	3	5	0.06
Ampicillin/Sulbactam	35	31	0.23
**Cefepime**	**25**	**12**	0.0001*
Cefotaxime	25	28	0.48
Amikacin	24	22	0.52
Ceftazidime	27	29	0.49
**Ceftriaxone**	**39**	**31**	0.05*
**Cefuroxime**	**14**	**31**	0.0001*
Cefuroxime axetil	3	2	0.69
**Ciprofloxacin**	**19**	**11**	0.0006*
**Ertapenem**	**4**	**2**	0.03*
Gentamicin	22	19	0.24
Meropenem	30	35	0.06
Nitrofurantoin	3	5	0.06
TMP/SXT	3	5	0.06
*Pseudomonas aeruginosa*			
**Amikacin**	**37**	**26**	0.0003*
**Cefepime**	**37**	**21**	0.0001*
**Ceftazidime**	**65**	**24**	0.0001*
**Ciprofloxacin**	**43**	**30**	0.0001*
**Gentamicin**	**29**	**13**	0.0001*
Meropenem	39	36	0.36

% R, Resistance percentage; Streptomycin-H y Gentamicin-H, High-level resistance to aminoglycosides. ^φ^For daptomycin, *S. aureus* isolates are classified as non-susceptible. *p ≤ 0.05 indicates statistical difference (bold Font). TMP/SXT, Trimethoprim/sulfamethoxazole.

#### 3.4.2 Staphylococcus aureus

**Table 3** shows that resistance to fluoroquinolones, with *S. aureus* followed two totally opposite paths. While for ciprofloxacin the percentages of resistance decreased, for moxifloxacin increased. Resistance to clindamycin and trimethoprim/sulfamethoxazole were significantly reduced, the former being the one that decreased the greatest proportion, while the percentages of resistance to linezolid have remained at the same levels.

#### 3.4.3 Klebsiella pneumoniae

Significant differences can be seen in the decrease of resistance percentages to cefepime, cefuroxime, and meropenem (**Table 3**), as well as a significant increase in ciprofloxacin.

#### 3.4.4 Acinetobacter baumannii

In 2014-2015, all *A. baumannii* isolates were resistant to cefotaxime, however, in 2019-2020 an abrupt decrease to 47% was observed (**Table 3**); also, a significant decrease in meropenem resistance is observed. Similar results in the percentages of resistance were detected for other third generation cephalosporins such as ceftazidime. Moreover, almost unchanged resistance percentages are observed specifically for ceftriaxone.

#### 3.4.5 Pseudomonas aeruginosa

From 2014-2015 to 2019-2020, a statistically significant decrease in the percentages of resistance to all the antibiotics evaluated for *P. aeruginosa* was detected, except for meropenem (**Table 3**). The marked decrease in the percentages of resistance to cephalosporins and fluoroquinolones stands out.

#### 3.4.6 Enterobacter cloacae

The decrease in the percentages of resistance to cephalosporins in *E. cloacae* from the period 2014-2015 to the period 2019-2020 is significant (**Table 3**). Resistance to gentamicin decreased in such a way that few isolates are reported as resistant to said antibiotic. On the other hand, the percentage of resistance to trimethoprim/sulfamethoxazole decreased notably.

## 4 Discussion

At the “José Eleuterio González” University Hospital in Monterrey, epidemiological surveillance of ESKAPE group microorganisms was carried out (Llaca-Díaz et al., 2013), and the prevalence values obtained by the authors did not coincide with those of the present study, as can be seen in **Figure 1**. In Hermosillo *A. baumannii* represented 7.4%, and in Ciudad Obregón 17% of the isolates, while in Monterrey it was 24.5% of the isolates and the first place within the microorganisms belonging to the ESKAPE group. Those differences could be attributable to the fact that the Monterrey study included only one health institution, while in thie present work 11 and three health units from Hermosillo and Ciudad Obregón, respectively, were included. The same was observed for *K. pneumoniae*, which represented 17.5% of the isolates in Monterrey, while in Hermosillo and Ciudad Obregón 29.0%. On the contrary, for *A. baumannii* and *K. pneumoniae*, the values are very similar to those of *P. aeruginosa* (22.1%), *S. aureus* (22.0%), *E. faecium* (3.2%) and *Enterobacter* spp. (10.5%) in comparison with Hermosillo and Ciudad Obregón, with values of 26.5% for *P. aeruginosa*, 22.6% for *S. aureus*, 2.0% for *E. faecium*, and 9.6% for *Enterobacter* spp. These results highlight the need to carry out multicenter studies of antibiotic resistance surveillance.

Similar studies have been carried out in some countries such as Brazil, where there are differences in the *K. pneumoniae* isolates, representing 41.0%, and *P. aeruginosa*, which represented 14.0%. Instead, *S. aureus* represented 22.0%, *Enterobacter* spp. 11.0%, *A. baumannii* 8.0% and *E. faecium* 4.0% (Silva et al., 2017). These results are similar to those detected in Hermosillo and Ciudad Obregón, but quite different from a Romanian study, where *S. aureus* was the microorganism isolated in the highest proportion with 62.4%, followed by *K. pneumoniae* with 16.6%, *P. aeruginosa* with 13.1%, *Enterobacter* spp. with 6.4%, *A. baumannii* with 1.2% and, *E. faecium* with 0.12% (Arbune et al., 2021). These discrepancies could be attributable to multiple factors, like population attended in each health institution, the number of wards in each hospital (the Brazilian hospital doesn’t have an Intensive Care Unit), and the different distribution of pathogens and resistance profile of them worldwide.

### 4.1 Enterococcus faecium

#### 4.1.1 Antibiotic resistance in the 2019-2020 period

The reduction to 0% of high-level resistance to aminoglycosides could indicate the loss of enzymes that inactivate these antibiotics, which could be a direct consequence of the limitation in their use. The results of susceptibility to antibiotics in *E. faecium* are lower than those reported in other institutions in the country (Garza-González et al., 2019). Enterococci isolates resistant to ciprofloxacin are also resistant to moxifloxacin (Cercenado, 2011), therefore 30% of the isolates are resistant to all commercially available fluoroquinolones. There is a high percentage of resistance to linezolid, even above that observed in other national institutions (2.4%) (Garza-González et al., 2019). No resistance to aminoglycosides was detected, so it is certain that the isolates do not synthesize aminoglycoside modifying enzymes (Cercenado, 2011) and less aggressive antimicrobial therapeutic schemes can be opted. In most mexican health institutions, vancomycin resistant enterococci (VRE) are isolated within a 20 to 25% interval (Garza-González et al., 2019); the results obtained in this study are lower (**Table 2**). Hermosillo is at higher resistance levels (74%) than those shown in the study by Garza-González and colleagues (2019), where 47% was obtained (Arbune et al., 2021).

**Figure 2:**
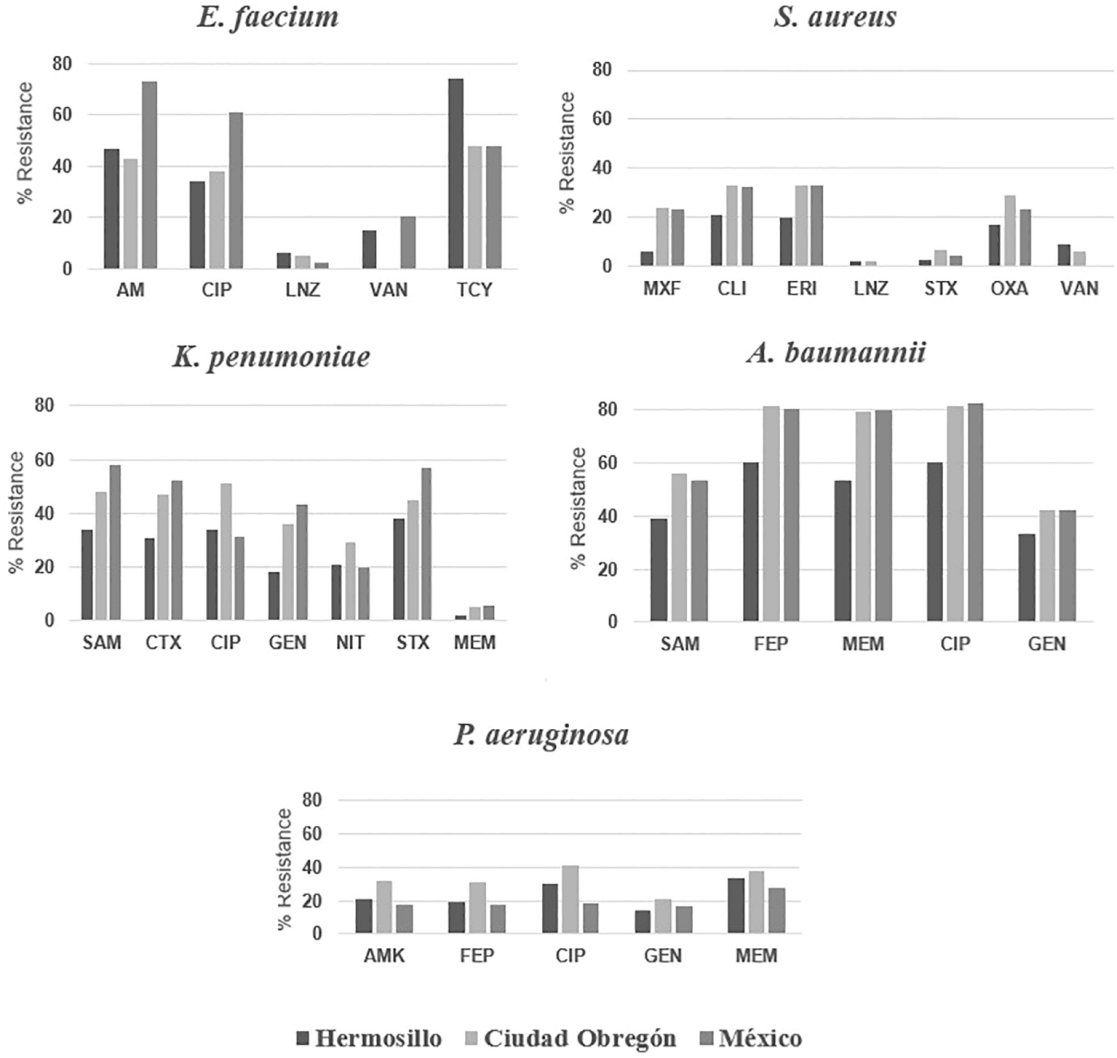
Comparative analysis of antibiotic resistance in isolates from ESKAPE organisms, in Hermosillo, Ciudad Obregón and other institutions in México. AMK, amikacin; AM, ampicillin; CIP, ciprofloxacin; LNZ, linezolid; TCY, tetracycline; MXF, moxifloxacin; CLI, clindamycin; ERI, erythromycin; OXA, oxacillin; VAN, vancomycin; SAM, ampicillin/sulbactam; CTX, cefotaxime; NIT, nitrofurantoin; GEN, gentamicin; FEP, cefepime; MEM, meropenem.

In general, *E. faecium* shows resistance percentages lower than what is observed in other health centers in the country (**Figure 2**). In 2018 studies carried out in Mexico, percentages of resistance to ampicillin of 73.2% were obtained (Garza-González et al., 2019). None of the resistance percentages obtained exceeds what is mentioned in the literature. A study carried out in Brazil found high percentages of resistance to vancomycin (79.2%), ampicillin (91.7%), ciprofloxacin (91.7%) and linezolid (8.3%) (Silva et al., 2017). **Table 2** shows a remarkably high value for resistance to tetracycline, a result opposite to that found by Silva and colleagues (2017) where most of the resistance values are excessively high, except for tetracycline (37.5%) (Llaca-Díaz et al., 2013). This shows a trend in health centers in Hermosillo, contrary to what was reported.

#### 4.1.2 Antibiotic resistance in the 2014-2015 and 2019-2020 periods

Even though there are reports indicating that vancomycin was the third most prescribed antibiotic in Mexican hospitals, during the 2016-2017 period (Miranda-Novales et al., 2020), the situation does not seem to affect the region since the increase of VRE isolates is minimal. In China, a surveillance study of antibiotic resistance was carried out in the pediatric population during the period of 2014-2017. This study reports an increase in the percentages of resistance to ampicillin between both periods (89.5%-91.7%) and a decrease in resistance to vancomycin (0.9%-0.3%) (Hui and Xiaolin, 2018), although, a slight increase in ampicillin and vancomycin resistance can be observed in **Table 3**, these results are not statically significative, but must be a situation to prevent the emergence of VRE to levels detected in other countries.

### 4.2 Staphylococcus aureus

#### 4.2.1 Resistance to antibiotics in the 2019-2020 period

The results in Ciudad Obregón were similar to other studies carried out in Mexico, where resistance levels of around 27% are reported (Garza-González et al., 2019). As shown in **Figure 1**, resistance to lincosamides and macrolides is lower in Hermosillo compared to Ciudad Obregón; in the latter, values close to other health institutions in Mexico are reported: 32.3% for clindamycin and 32.9% for erythromycin (Garza-González et al., 2019). The levels of resistance to macrolides and lincosamides are identical, observing the effect of resistance to MLS (De Oliveira et al., 2020), which suggests high levels of resistance to streptogramins in Ciudad Obregón.

Even though resistance to daptomycin and linezolid are low, they represent an alarm sign as they are drugs of last choice for the treatment of infections caused by MRSA (Guo et al., 2020). The resistance levels observed in Ciudad Obregón for trimethoprim/sulfamethoxazole are higher than Hermosillo. Given the resistance mechanism, it is possible that there is a plasmid-mediated dissemination (Foster, 2017). In Ciudad Obregón, the incidence of MRSA isolates is significantly higher than in Hermosillo. It is also true in relation to other health institutions in Mexico, where 23.1% is reported, as can be seen from the results from oxacillin resistance (Garza-González et al., 2019).

#### 4.2.2 Resistance to antibiotics in the 2014-2015 and 2019-2020 periods

The decrease in isolates not susceptible to daptomycin should be highlighted, which represents something positive for patients as it is an antibiotic used against MRSA (Guo et al., 2020). MRSA isolation can be verified at both periods. It is true that they are not comparable to the high levels reported in Ciudad Obregón, but it is still of great importance due to its high capacity to transmit its resistance mechanisms through plasmids (Foster, 2017) and the high mortality to invasive infections caused by MRSA (Turner et al., 2019). Arbune et al. (2021) reported that in a Romanian hospital MRSA isolation decreased from 39.6% in 2016 to 30.9% in 2018 and increased to 46.2% in 2020 (Arbune et al., 2021). In a Pakistani hospital it is observed how MRSA isolation was at 60% in mid-2018, decreased to 30% for the same period in 2019, and then followed by an increasing trend for the remainder of the year (Wadi-Al Ramahi and Jamal, 2020). These data are similar to those reported in **Table 3**, so it seems that there is a trend toward an increase (or fluctuation) in MRSA worldwide, (probably associated to the emergence of new clones as well as changes in the occurrence of some clonal lineages of MRSA) (Zarfel et al., 2023).

### 4.3 Klebsiella pneumoniae

#### 4.3.1 Resistance to antibiotics in the 2019-2020 period

The high levels of resistance to cephalosporins compared to other groups of antibiotics could have been favored by their high prescription rate in Mexican hospitals (Miranda-Novales et al., 2020). Compared with results from other Mexican regions, both cities considered in the present study are above from those reported for ciprofloxacin (31.1%) and nitrofurantoin (19.9%), but this study showed lower results for trimethoprim/sulfamethoxazole (56.8%) and gentamicin (43.5%) (Garza-González et al., 2019). As can be seen in **Figure 2**, the resistance detected for most of the antibiotics in *K. pneumoniae* are lower in Hermosillo than in Ciudad Obregón but are lower in Ciudad Obregón compared to other institutions in the country, except for ciprofloxacin and nitrofurantoin (Garza-González et al., 2019).

#### 4.3.2 Resistance to antibiotics in the 2014-2015 and 2019-2020 periods

The decrease in resistance to meropenem broadens the therapeutic options, avoiding the use of last-line antibiotics, such as colistin (Wyres et al., 2020). The decrease in resistance to gentamicin would allow the use of this antibiotic as a therapeutic alternative, in the management of infections caused by *K. pneumoniae* resistant to β-lactams. Likewise, having greater availability for its use will prevent the increase in resistance to β-lactams by reducing their use, especially in hospitalized patients. On the other hand, resistance to fluoroquinolones has increased significantly, most likely due to the increase in the consumption of ciprofloxacin and levofloxacin worldwide in recent years (Versporten et al., 2018). In China, the difference in the *K. pneumoniae* resistance patterns was studied during the 2008 to 2015 period. A decrease in the prevalence of ESBL-producing *K. pneumoniae* isolates was found, from 39.5% in 2008 to 21.5% in 2018. Contrary to this, there was an increase in the prevalence of carbapenem-resistant *K. pneumoniae*, from 2.5% in 2008 to 15.8% in 2015 (Hu et al., 2020). In Hermosillo, there were no statistically significant variations in resistance to β-lactams, but contrary to China, resistance to carbapenems decreased. A similarity between both studies is the increase in resistance to ciprofloxacin; in China it increased from 19.6% in 2014 to 24% in 2018 (Hu et al., 2020).

### 4.4 Acinetobacter baumannii

#### 4.4.1 Resistance to antibiotics in the 2019-2020 period

The results of resistance to cephalosporins and carbapenems are not typical at the health centers of Hermosillo and Ciudad Obregón, although in other hospitals in Mexico, resistance to cefepime stands at 80.3% and to meropenem at 79.6% (Garza-González et al., 2019). As observed for *K. pneumoniae*, resistance may be favored using β-lactams in Mexican health institutions (Miranda-Novales et al., 2020). Compared to other Mexican cities, where resistance to ampicillin/sulbactam stands at 53.2%, it is slightly lower in Ciudad Obregon and notably lower in Hermosillo with 39% (Garza-González et al., 2019). Resistance to fluoroquinolones is favored by the increase in their consumption worldwide (Versporten et al., 2018). The results of this work and of previous studies suggest that there is a generalized problem of *A. baumannii* MDR (**Figure 2**). Although Hermosillo presents the lowest percentages of clinical isolates of *A. baumannii* resistant to most of the antibiotics evaluated, the possibility of MDR isolates in this city should not be ruled out. The high levels of resistance reaffirm the great capacity of *A. baumannii* to evade antibacterials and how limited these health centers find themselves when they face infections by this agent. In Monterrey, Llaca-Díaz et al. (2013) found 85% of isolates resistant to ceftriaxone and ceftazidime, 86% to ciprofloxacin, 75% to meropenem, and 87% to trimethoprim/sulfamethoxazole (Llaca-Díaz et al., 2013). These results are agreed those reported in this study for Ciudad Obregón, indicating that there could be a similarity between the resistance profiles for *A. baumannii* with that study.

#### 4.4.2 Resistance to antibiotics in the 2014-2015 and 2019-2020 periods

Although there is no factor related to the results obtained, ceftriaxone is the most used cephalosporin worldwide in recent years (Versporten et al., 2018) so it would not be surprising if resistance to it had been maintained and even increased. On the other hand, it is possible to identify a decrease in the percentages of resistance to carbapenems, which is positive for patients of these institutions, especially those hospitalized. Wadi-Al Ramahi et al. (2020) found that in the period from 2014 to 2019 the prevalence of carbapenem-resistant *A. baumannii* isolates increased from 96% to 100% in Pakistan (Wadi-Al Ramahi and Jamal, 2020), contrary to what was detected in the present work, where a significant decrease was observed. These results could be partially attributable to the low number of *A. baumannii* clinical isolates, recovered in Hermosillo health units (24 in 2014-205 and 79 in 2019-2020) and to differences in antibiotic management between both countries.

### 4.5 Pseudomonas aeruginosa

#### 4.5.1 Resistance to antibiotics in the 2019-2020 period

Resistance to ceftazidime and cefepime in Ciudad Obregón should be considered important since these drugs have good bactericidal activity against *P. aeruginosa* (Hilal and Brunton, 2015). Therefore, it is advisable to evaluate whether the high resistance that is occurring is due to the resistance of the bacteria itself, or to bad practices in the use or prescription of antibiotics. Gentamicin seems to be the most effective therapeutic option against *P. aeruginosa* both in Ciudad Obregón and in Hermosillo, but as previously mentioned, it presents toxicological characteristics that could restrict its administration. In other cities of Mexico, 18% of *P. aeruginosa* isolates are resistant to ciprofloxacin (Garza-González et al., 2019), therefore, the results found in the present study are greater than those reported in the rest of the country. Various studies show that the resistance profiles of *P. aeruginosa* vary depending on the clinical sample. In Latin America, 39% of *P. aeruginosa* isolates obtained from patients with UTIs are resistant to fluoroquinolones (Karlowsky et al., 2017). These values are close to what was obtained for Hermosillo and Ciudad Obregón, so it is possible, and considering that the urine samples were the predominant ones in this study, that this same situation is occurring in Hermosillo. On the other hand, in the study by Garza-González et al. (2019), 36% showed resistance to this family of antibiotics (**Figure 2**).

Similarly, to what was observed for other ESKAPE group microorganisms, high percentages of resistance to cephalosporins and fluoroquinolones are detected in comparison with other members, and the proposed explanation remains the same: the wide prescription of these antibiotics at a national and global level (Miranda-Novales et al., 2020; Versporten et al., 2018).

#### 4.5.2 Resistance to antibiotics in the 2014-2015 and 2019-2020 periods

The decrease in the percentages of resistance to cephalosporins and fluoroquinolones is important for their optimal therapeutic options, because it has good absorption, low toxicity, and good antipseudomonal activity, key aspects for the incarcerated patient (Mezzatesta et al., 2012). In a hospital with infectious diseases in Romania, an increase in the prevalence of carbapenem-resistant *P. aeruginosa* was detected from 9% in 2016 to 60.8% in 2018 (Hilal and Brunton, 2015). It should be noted that Hermosillo did not show an increase in the number of isolates of this type.

### 4.6 Enterobacter cloacae

#### 4.6.1 Resistance to antibiotics in the 2019-2020 period

Resistance to carbapenems is low because the production of carbapenemases by this bacterium is rare, although it has increased in recent years. The difference between resistance to ertapenem and meropenem is due to little-studied genetic factors in *E. cloacae* (Mezzatesta et al., 2012). The appearance of resistance to nitrofurantoin is associated with specific mutations in coding genes (Huttner et al., 2015), therefore, it is possible that in hospitals in Ciudad Obregón the resistance is given by clones of the same strain that in theory would possess the same mutation. Like most members of the ESKAPE group, the high resistance to cephalosporins and fluoroquinolones is favored by their high and growing consumption (Miranda-Novales et al., 2020; Versporten et al., 2018).

#### 4.6.2 Resistance to antibiotics in the 2014-2015 and 2019-2020 periods

This organism is known for β-lactamase synthesis, however, a decrease in resistance to cephalosporins is observed (Mezzatesta et al., 2012). As mentioned before, the use of β-lactams represents a good therapeutic option so that this decrease has a direct positive impact on therapeutic options for patients. Resistance to gentamicin decreased in such a way that few isolates are reported as resistant to said antibiotic. This provides an important therapeutic option in *E. cloacae* isolates resistant to available antibiotics. On the other hand, the percentage of resistance to trimethoprim/sulfamethoxazole decreased notably, so it could begin to be considered as an important treatment in urinary tract infections (Hilal and Brunton, 2015), expanding the therapeutic options. Liu et al. (2021) carried out an assessment of the prevalence of *E. cloacae* complex isolates in a hospital in China (Liu et al., 2021). The authors detected an increase from 2.5% in 2010 to 11.9% in 2018 and mentioned that increased carbapenem resistance worldwide must be associated to their widely used in the treatment bacterial infections, mainly those caused by MDR Gram-negative.

## 5 Conclusions

During the 2019-2020 period, 4,545 isolates of ESKAPE microorganisms were recovered, in eight health institutions in Hermosillo and three in Ciudad Obregón, Sonora. The percentage of isolates showing resistance to studied were higher in Ciudad Obregón than in Hermosillo, and probably is associated with the population attended by different health institutions (primary care attention or reference hospitals). Also, in Hermosillo, some health institutions participated in previous surveillance studies, and the previous results could have been supported some institutional policies of antibiotic prescription. *Klebsiella pneumoniae* was the microorganism with the highest number of isolates identified in these 11 health institutions followed by *Pseudomonas aeruginosa* and *Staphylococcus aureus*. In Hermosillo, isolates of *Enterococcus faecium* showed high levels of resistance to tetracycline, while in Ciudad Obregón no isolates of vancomycin-resistant enterococci were identified. Most of the percentages of resistance to antibiotics in *S. aureus* are statistically significant and higher in Ciudad Obregón than in Hermosillo. In Ciudad Obregón there is a high prevalence of MRSA and *S. aureus* isolates resistant to erythromycin, clindamycin, and fluoroquinolones. A decrease in susceptibility to daptomycin is reported in health institutions of both cities. *K. pneumoniae* showed high levels of resistance to cephalosporins, ciprofloxacin and nitrofurantoin, but lower for trimethoprim/sulfamethoxazole and gentamicin. The resistance detected for most of the antibiotics in *K. pneumoniae* were lower in Hermosillo than in Ciudad Obregón. *Acinetobacter baumannii* presents high percentages of resistance to all antibiotics in Ciudad Obregón, including carbapenems. For *P. aeruginosa*, except for meropenem, all resistance percentages are higher in Ciudad Obregón than in Hermosillo, and a high prevalence of carbapenem-resistant *P. aeruginosa* was detected. *Enterobacter cloacae* presents high percentages of resistance to β-lactams, except for carbapenems.

In six health institutions in Hermosillo, *E. faecium* decreased its resistance to fluoroquinolones and aminoglycosides from the 2014-2015 to 2019-2020 periods, however, resistance to linezolid was detected in the latter. Resistance to trimethoprim/sulfamethoxazole and clindamycin decreased significantly, but the number of MRSA and vancomycin-resistant *S. aureus* isolates increased. Ciprofloxacin-resistant *S. aureus* isolates decreased, but the number of moxifloxacin-resistant isolates increased. Resistance to cefepime, meropenem, and gentamicin decreased in *K. pneumoniae*, while percentages of resistance to fluoroquinolones increased between 2014-2015 and 2019-2020. The isolates of *A. baumannii* presented a decrease in the percentages of resistance to cefotaxime and meropenem. *Enterobacter cloacae* presents high percentages of resistance to β-lactams, except for carbapenems; however, a decrease in the percentages of resistance to β-lactams, aminoglycosides, and trimethoprim/sulfamethoxazole was detected in 2019-2020.

The differences between health institutions between two major cities of the State of Sonora, that are geographically close (157 miles), support the worldwide recommendations to maintain an active epidemiological surveillance program that allow the knowledge of local antibiotic resistance. Furthermore, differences in antibiotic resistance should be monitored to improve empiric treatments, according to the epidemiological information from each health institution, prevent the spread of MDR bacteria, and avoid their resistance to a limited options of antibiotics available at this moment.

## Data availability statement

The raw data supporting the conclusions of this article will be made available by the corresponding authors, without undue reservation.

## Author contributions

MÁ-A: Conceptualization, Writing – original draft. PF-C: Data curation, Formal analysis, Writing – review & editing. ER-B: Writing – review & editing. LC-C: Supervision, Writing – review & editing. IQ-R: Supervision, Writing – review & editing. LD-Z: Supervision, Writing – review & editing. EB-M: Conceptualization, Investigation, Methodology, Writing – original draft, Writing – review & editing.
